# METABOLOMICS OF LONGEVITY AND LIFESPAN

**DOI:** 10.1093/geroni/igad104.2056

**Published:** 2023-12-21

**Authors:** Paola Sebastiani, Nalini Raghavachari

## Abstract

Serum metabolomics has been an important source of biomarkers of aging and longevity for years. This symposium will bring together investigators from large studies of human longevity to provide an overview of recent discoveries on serum metabolomics of aging and extreme human longevity, their connections to genetic variations, and highlight the challenges of correlating metabolomic profiles of aging in human studies and across multiple species. Dr. Sebastiani will describe results from analyses of serum metabolomics of participants enrolled in the Long Life Family Study, highlight similarities and differences between metabolomic profiles of old age and extreme old age, and some connections with genetics of extreme human longevity. Dr. Rappaport will connect specific variations of the APOE alleles to metabolomic profiles and describe a possible role of bioenergetics pathways in mediating the effect of APOE to longevity and resistance to Alzheimer’s disease. Dr. Monti will expand the characterization of metabolomics of aging and extreme human longevity in a large metabolomic study of very old centenarians by using traditional statistical analyses and novel machine learning techniques. His analysis identifies rich signatures of aging and longevity that include well known metabolites and point to bile acids and several classes of steroids as important marker of longevity. Analytical innovations will be taken further by Dr. Schork who will introduce a novel approach based on distance of profiles to analyze multiple metabolites simultaneously and show the value of this approach to analyze metabolomic profiles of maximum lifespan across multiple species. This is a Geroscience Interest Group Sponsored Symposium.

